# Supplementary material for: Concurrence of form and function in developing networks and its role in synaptic pruning
Source: Nat Commun. 2018 Jun 8;9:2236. doi: 10.1038/s41467-018-04537-6 (PMC5993834; doi:10.1038/s41467-018-04537-6)
Supplement: Supplementary file 1 — Supplementary Information [file 41467_2018_4537_MOESM1_ESM.pdf]

Supplementary Information for "Concurrence of form and function in developing networks and its role in synaptic pruning"

Ana P. Millán[a*] et al.

April 23, 2018

Supplementary Figures

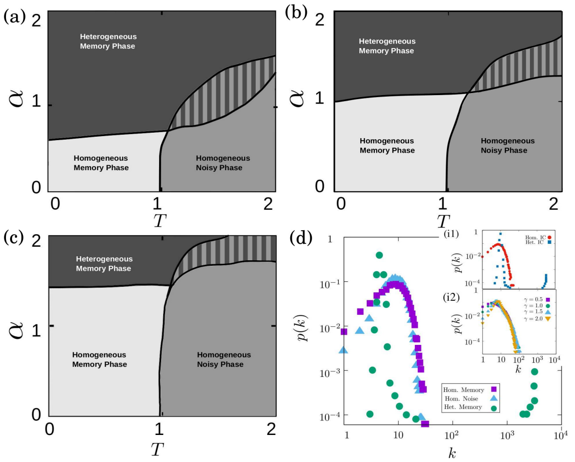

Supplementary Figure 1: **Parameter analysis.** Phase diagrams of the system for $\gamma = 0.5$, 1.0 and 1.5 respectively in panels a,b,c; and for $n = 10$, $\kappa_0 = 20$, $\kappa_\infty = 10$ and $N = 1600$. Results for $\gamma = 1.0$ hold qualitatively for other values, but the region corresponding to each phase depends on γ. Due to the structure-memory coupling, the critical value α_c is depends slightly on the temperature, as shown in the main paper for $\gamma = 1.0$. Data has been averaged over 20 realizations. Panel **(d)** shows $p_\infty(k)$ in some representative cases. `Main plot:` Three (T, α) points corresponding respectively to the homogeneous memory $(0.5, 0.5)$, homogeneous noise $(1.5, 0.5)$ and heterogeneous memory $(0.5, 1.5)$, as indicated in the caption. The homogeneous distributions are fairly similar, whereas the heterogeneous one is bimodal. `Inset i1:` Comparison between homogeneous (red circles) and heterogeneous (blue squares) IC in the bistability region $(T = \alpha = 1.5)$. Homogeneous IC fall into the noisy homogeneous phase, whereas heterogeneous ones maintain memory and organize into a bimodal distribution. `Inset i2:` Examples of $p_\infty(k)$ along the critical transition $\alpha_c^t(T)$ for $T = 0.5$ and different values of γ, as indicated in the caption. Results are are mostly independent on γ, showing a heterogeneous and roughly scale-free behaviour, but given the finite size of the system the exponent cannot be measured. Data averaged over 100 realizations.

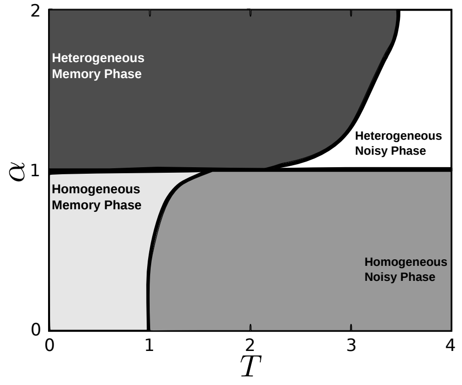

Supplementary Figure 2: **Topological limit** Here we include an analysis of the phase diagram of the system in the limit $I_i \to k_i$ (figure **??**). Without the physiology-structure coupling, network structure is simply determined by α and, this, in turns, characterizes the memory transition. In this way, for $\alpha < 1$, networks are homogeneous, and there is a continuous transition from memory to noise. The critical temperature for this transition moves from $T_C = 1$ for completely homogeneous networks (so that $p_\infty(k) = \delta_{k,k_0}$) for $\alpha \ll 1$, to higher T as the nodes degrees gain some heterogeneity, according to previous studies [1]. On the other hand, for $\alpha > 1$ and a finite-size system ($N = 1600$ in the results shown), the temperature of the transition keeps growing with α. In the thermodynamic limit, the transition would take place at infinite temperature according to the literature [2]. Notice that this diagram has been extended up to $T = 4$ in order to appreciate the transition for $\alpha > 1$. In conclusion, as a result of the lack of feed-back from the physiology, the bistability region disappears, since the structure of the network does no longer depend on the memory state. Phase diagram of the model in the topological limit. Data: $N = 1600$, $\gamma = 1$, $\kappa_0 = 20$, $\kappa_\infty = 10$, $n = 5$.

References

[1] Torres, J. J, Muñoz, M. A., Marro, J. & Garrido, P. L. Influence of topology on the performance of a neural network. *Neurocomputing* **58**, 229-234 (2004).

[2] Leone, M., Vázquez, A., Vespignani, A. & Zecchina, R. Ferromagnetic ordering in graphs with arbitrary degree distribution. *Eur. Phys. J. B.* **28**, 191-197 (2002).
